# Ischemic Monomeric Neuropathy in a Woman with Sickle Cell Anaemia

**DOI:** 10.1155/2016/8628425

**Published:** 2016-11-22

**Authors:** Alexandra Agapidou, Laura Aiken, Lisa Linpower, Dimitris A. Tsitsikas

**Affiliations:** Haemoglobinopathy Service, Department of Haematology, Homerton University Hospital NHS Foundation Trust, London, UK

## Abstract

Sickle cell disease is an inherited haemoglobinopathy that can affect multiple organs and systems. The most common neurological complication in sickle cell disease is stroke and silent cerebral infarcts. Peripheral nervous system involvement has been described but is exceedingly rare. Herein, we describe the case of a young woman who presented with acute flaccid paralysis and sensory loss of the left lower extremity in the context of a painful vasoocclusive crisis which resolved rapidly after receiving an emergency automated red cell exchange transfusion.

## 1. Introduction

Sickle cell disease (SCD) is a common hemoglobinopathy that may affect every human organ or system [1]. Nervous system involvement is one of the most devastating aspects of the disease. Both central (CNS) and peripheral (PNS) nervous system complications have been described in the literature with CNS involvement being by far the most common [2]. Acute neurological presentations include acute ischemic or hemorrhagic stroke [3], infections, posterior reversible encephalopathy syndrome [4], central sinus venous thrombosis [5], and peripheral mononeuropathy [6]. Diagnosis of acute neurological events in patients with SCD requires rapid access to appropriate imaging and management requires a multidisciplinary approach involving haematologists, neurologists, specialist radiologists, and stroke-rehabilitation physicians. We report the presentation, management, and outcome of a young woman with HbSS who developed acute paralysis and sensory loss of the left lower extremity in the context of a painful vasoocclusive crisis (VOC). The patient gave consent to the publication of this report.

## 2. Case Report

The patient, a 19-year-old woman with sickle cell anaemia, was admitted due to acute onset of severe sensory-motor impairment of her left lower extremity. The patient was awakened during the early morning hours by severe pain in the waist, typical of a VOC, and realized that she had decreased sensation and mobility on her left leg (characteristically mentioned that she could not feel or move the affected leg). Mobility and sensation in the right lower and both upper extremities were intact. Her mental status was normal, her speech was unaffected, and there were no sphincter disturbances. Neurological examination at the time of admission revealed power 1-2 of all muscle groups of the affected limb. Tone was reduced and deep tendon reflexes were absent even after reenforcement, suggesting peripheral nerve pathology. Sensation was subjectively reported by the patient as being 80% decreased compared to normal and proprioception was lost. Gait could not be assessed as the patient could not stand. Save for lower spinal and left hip tenderness on palpation in keeping with VOC, the rest of the neurological and general physical examination was unremarkable. Results of baseline diagnostic tests are summarized in Table 1. Computer tomography (CT) of the brain, magnetic resonance imaging with angiography (MRI/MRA) of brain, and circle of Willis and MRI of the spine were performed. CT of the brain was unremarkable with no evidence of intracranial hemorrhage and subacute or any established territorial infarct. MRI of the brain showed no evidence of acute ischemia and there were no vascular abnormalities on MRA. MRI of the spine showed normal cord caliber and signal, with no evidence of intramedullary infarcts. There was no evidence of hemorrhage or compression.

Conservative therapy with fluids and parenteral opiate analgesia led to improvement of pain. In the absence of any anatomical abnormalities, it was felt that the most likely cause for her acute peripheral nerve injury was ischemia due to vasoocclusion and the patient underwent emergency automated red cell exchange transfusion (ARCET) 24 hours after her initial presentation. She received 12 units of red cells as per UK national standards for patients with SCD [7] using the Spectra Optia Apheresis System. Pre- and posttransfusion haematological values are summarized in Table 2.

ARCET led to immediate clinical improvement with almost complete resolution of the neurological deficit by the end of the procedure and, shortly afterwards, the patient was able to walk independently. She was reviewed again by the neurologists who confirmed complete resolution of the deficit. Even though the patient was scheduled to have nerve conduction studies and a diagnostic lumbar puncture, these were now considered unnecessary and cancelled. She was discharged home, 48 hours after admission, without any neurological deficit.

## 3. Discussion

SCD is an autosomal recessive inherited haemoglobinopathy, resulting from the substitution of glutamate by valine in position 6 of the *β* globin gene giving rise to an abnormal haemoglobin or “sickle haemoglobin” (HbS) [8]. HbS molecules within the red cell tend to polymerize under certain conditions such as hypoxia, acidosis, or dehydration giving rise to the characteristic “sickle” shape of the erythrocytes. These deformed and rigid erythrocytes lead to the two cardinal manifestations of SCD: (a) vasoocclusion due to impaired rheology and infarction of the microvasculature and (b) haemolysis and chronic anaemia due to red cell deformability and increased destruction both intra- and extravascularly [9]. Intravascular sickling and haemolysis lead to a cascade of events including endothelial damage, ischemia re-perfusion injury, alteration of adhesion molecules, activation of coagulation, and nitric oxide (NO) depletion, that result in the systemic vasculopathy and chronic inflammatory state that characterize SCD [9].

Neurological complications involve principally the brain, while the spinal cord or the PNS are rarely involved [10]. Nerve distribution varies and some nerves are more sensitive to ischemia than others [11–13]. Size and number of nutrient vessels that supply the different peripheral nerves are different among individuals and among nerves in the same individual. These variations make certain individuals and certain nerve regions even in the same individual more susceptible when exposed to ischemia [14]. Animal studies have found the proximal tibial and peroneal nerves, distal sciatic nerve, and distal foot nerves to be most vulnerable to ischemia [14]. In humans, the peroneal, sural, tibial, ulnar, median, and radial nerves are most frequently affected by ischemia [11–13]. The extensive peripheral nerve vascular supply and the widespread network of large capillaries of the peripheral nerves could possibly explain the reason why ischemic neuropathy is rare in SCD [14].

There are limited reports in the literature of acute peripheral neuropathies presenting in patients with SCD. One such reports a mononeuropathy affecting the median nerve presenting as a painful VO [6]. They conclude that the neurophysiology confirmed an ischemic injury to the median nerve with the only identifiable cause being that of a vasoocclusive insult. The authors acknowledge that PNS involvement is rare but the individual variation in nervous system anatomy and vascular supply mean this is still possible.

A further case report describes two patients who developed an acute mononeuritis multiplex in the context of a VOC [6]. One went on to be diagnosed with a mixed connective tissue disease (MCTD) alongside their SCD. It the other patient, similar to our case, no other aetiology could be identified and all autoimmune tests were negative. At three-year follow-up, this patient had some mild residual weakness in the foot, and no alternative diagnosis had made its self-known. Neurophysiology confirmed both cases were the result of nerve ischemia and, despite the MCTD diagnosis, they attributed the ischemia to SCD. In both patients, symptoms developed in the lower limb, as the case in our patient and as previously discussed, nerves in the lower limb are more prone to ischemic insult.

Other problems seen in a sickle cell crisis such as acidosis and dehydration are further risk factors for developing nerve ischemia. These factors, together with a problematic vascular supply of the nerve, could lead to development of nerve ischemia in the context of a sickle cell crisis. In the acute settling, all three patients were treated conservatively with analgesia and hydration. Although there was some level of neurological improvement over time, the patients were left with residual neurological deficits.

In the case we describe, the acute peripheral sensory-motor deficit in the context of a painful VOC with absence of any abnormal radiological findings of the brain and spine was most likely the result of peripheral nerve ischemia due to vasoocclusion. This is further supported by the rapid reversal of the clinical symptoms and signs after ARCET. In the previous cases described, individual nerves were the cause of the neurological deficit. Although we could not confirm the location of the lesion due to the swift reversal of symptoms, the presentation, along with the examination findings, would support a lumbosacral plexopathy.

There are few limitations in our diagnostic approach due to the absence of nerve conduction studies and examination of the cerebrospinal fluid to exclude other pathologies, such as the Guillain–Barre syndrome. The patient's swift recovery made the above procedures clinically unjustifiable. To our knowledge, this is the first case of acute peripheral nerve injury in SCD treated successfully with ARCET.

## Figures and Tables

**Table 1:** Baseline haematological, biochemical, immunological, and microbiological investigations.

Parameter	Value	Normal ranges
Sodium	138	135–147 mmol/L
Potassium	3.8	3.4–4.9 mmol/L
Urea	2.2	2–6.6 mmol/L
Creatinine	57	60–100 umol/L
Hb	80	115–165 g/L
Platelets	117	150–400*∗*10^9^/L
WBC	7.9	4–11*∗*10^9^/L
CRP	<5	<5 mg/L
ANA	Negative	
Liver/kidney micrososmal Abs	Negative	
Mitochondrial Abs	Negative	
Smooth muscle Abs	Negative	
Gastric parietal cell Abs	Negative	
DNA Ab (IMF)	0.9	1–10 iu/Ml
B19 parvovirus IgG	Not detected	
B19 parvovirus IgM	Not detected	
*Haemophilus influenzae* Abs	>5	1–1000
Pneumococcal IgG total	>270	0.3–1000
EBV IgM	Not detected	

Hb: haemoglobin, WBC: white blood count, CRP: C-reactive protein, ANA: antinuclear antibodies, Abs: antibodies, IMF: immunofluorescence, and EBV: Epstein–Barr virus.

**Table 2:** Haematological values before and after exchange transfusion.

	Pre-ARCET	Post-ARCET
Hb (g/L)	80	85
Hct	0.248	0.251
HbA%	4.7	69.2
HbS%	86	16.5

ARCET: automated red cell exchange transfusion; Hct: haematocrit.
